# Instrumented Functional Mobility Assessment in Elderly Patients Following Total Knee Arthroplasty: A Retrospective Longitudinal Study Using the Timed Up and Go Test

**DOI:** 10.3390/life15091409

**Published:** 2025-09-07

**Authors:** Andrei Machado Viegas da Trindade, Leonardo Pinheiro Rezende, Helder Rocha da Silva Araújo, Rodolfo Borges Parreira, Claudia Santos Oliveira

**Affiliations:** 1 Department of Orthopedics Surgery, Municipal Hospital of Aparecida de Goiânia (HMAP), Aparecida de Goiânia 74936-600, Brazil; andrei.trindade@einstein.br; 2 Health Sciences Program, Santa Casa de São Paulo School of Medical Sciences, São Paulo 01221-020, Brazil; csantos.neuro@gmail.com; 3 Faculty of Medicine, University Evangelica of Goiás, Anápolis 75083-515, Brazil; 4 Department of Orthopaedics and Traumatology, Federal University of Goiás (UFG), Goiânia 74605-050, Brazil; drhelderrocha@hotmail.com; 5 Human Movement Analysis Laboratory, University Evangelica of Goiás, Anápolis 75083-515, Brazil; rodolfo.fcmscsp@gmail.com

**Keywords:** arthroplasty, replacement, knee, gait, accelerometry, timed up and go test, rehabilitation

## Abstract

In the context of the rising demand for total knee arthroplasty (TKA) in older adults and persistent uncertainty about the quality of long-term functional recovery, this study evaluated elderly patients’ mobility after unilateral TKA via a transquadriceps approach using instrumented Timed Up and Go (TUG) tests. A total of 20 patients treated between 2022 and 2024 at a tertiary hospital were invited to participate in this observational, retrospective, descriptive study, and 19 met the inclusion criteria (age 50–80 and Kellgren–Lawrence ≥ 4). The participants performed two TUG trials at two postoperative time points (18 and 53 months), with an inertial measurement unit (G-sensor) capturing 15 kinematic variables. When comparing the postoperative time points, it was found that the total TUG duration remained stable (14.97 ± 3.48 vs. 15.47 ± 2.93 s; *p* = 0.58), while the mid-turning peak velocity increased significantly (106.44 ± 30.96 vs. 132.77 ± 30.82°/s; *p* = 0.0039; r = 0.88). The end-turning velocity and sit-to-stand parameters showed small-to-moderate effect size gains without statistical significance. These findings suggest that, in the first year following surgery, patients continue to experience difficulties with movement fluidity and motor control—especially during turning—underscoring the value of segmented, sensor-based assessments and the need for extended rehabilitation protocols that emphasize rotational control and balance. These findings provide clinically relevant parameters that can support future interventional studies and help guide rehabilitation planning after TKA.

## 1. Introduction

Osteoarthritis (OA) is a degenerative joint disease that predominantly affects older adults and is the leading indication for total knee arthroplasty (TKA) [1]. Its prevalence increases with age, and most patients undergo TKA between 60 and 80 years of age [2]. Advanced degenerative or inflammatory disease is commonly treated by TKA, an orthopedic procedure that is both highly prevalent and effective for end-stage OA, providing pain relief and improving quality of life, particularly in older adults. Nevertheless, long-term functional recovery often fails to reach the levels observed in age-matched healthy individuals. When performed via the transquadricipital (medial parapatellar) approach, TKA requires a longitudinal incision through the quadriceps tendon to expose the joint [3]. The principal advantage of this traditional quadriceps-splitting medial parapatellar technique is its familiarity to surgeons and the excellent exposure it affords, facilitating intraoperative visualization and manipulation.

TKA is among the most successful and commonly performed orthopedic procedures worldwide, principally in individuals over 60 years of age to treat advanced OA [4]. Demand for TKA is projected to increase substantially as populations age and the prevalence of degenerative joint disease rises [5]. A similar upward trend has been observed in Brazil, where the number of TKAs performed by the Unified Health System (SUS) within this age group has grown [6].

Despite well-established benefits of TKA, gaps remain in the comprehensive understanding of functional recovery in older adults, particularly with respect to movement quality and asymmetries between the operated and non-operated limbs [7]. Recent studies using wearable sensors such as IMUs indicate that, even after conventional rehabilitation, subtle deficits persist in gait biomechanics, dynamic postural control during functional tasks, and the ability to perform rapid, safe changes of direction [5,8]. Therefore, detailed characterization of these residual impairments and their associations with different surgical techniques and rehabilitation approaches remains necessary to optimize long-term outcomes [9].

Violation of the extensor mechanism inherent to the transquadricipital approach frequently leads to postoperative quadriceps weakness, which impairs functional mobility and manifests as difficulty with ambulation and rising from a chair [10]. Accurate evaluation of these functional deficits, therefore, requires methods that capture movement quality in addition to simple timing measures. Instrumenting the Timed Up and Go (TUG) test with an inertial measurement unit (IMU) enables quantitative, segmented analysis of performance, providing parameters such as acceleration profiles and angular smoothness during sit-to-stand transitions and turning maneuvers [11,12]. This objective, sensor-based diagnostic approach is essential for detecting subtle limitations and for guiding more targeted rehabilitation strategies in older adults following arthroplasty [13].

Given this persistent gap in knowledge about the quality of motor recovery, the present study asks: what is the functional performance of elderly patients following unilateral TKA via the transquadricipital approach when evaluated quantitatively with an IMU during the TUG test? The study’s relevance derives from employing objective, high-resolution metrics to characterize deficits not captured by conventional timed assessments. Although the transquadricipital approach is widely used, few investigations have applied IMU-based analyses to objectively evaluate long-term post-surgical outcomes and to inform more specific rehabilitation protocols. In particular, sensor-based analyses of complex movements such as turning in elderly TKA patients remain scarce. Accordingly, we assessed functional mobility patterns in older adults after TKA using instrumented TUG testing at two postoperative time points. We hypothesized that certain kinematic parameters—especially those related to turning and transitional movements—would improve over time, whereas overall task duration might remain stable.

## 2. Materials and Methods

This is an observational, retrospective, and descriptive study conducted with patients who underwent unilateral total knee arthroplasty (TKA) via the transquadricipital surgical approach, aimed at identifying and describing those who were operated on using this technique.

Initially, the target population comprised 19 patients who underwent total knee arthroplasty (TKA) at a tertiary hospital in Goiás, Brazil, between 2022 and 2024. All patients underwent primary TKA under spinal anesthesia via a transquadricipital approach at the same hospital and by the same surgical team; all received implants from a single manufacturer, were managed by the same inpatient physical therapy team, and adhered to a standardized postoperative rehabilitation protocol. The sample size calculation was based on the findings of Pfeufer et al. (2022) [14], who observed a mean increase of 0.27 m/s in gait speed (from 0.75 to 1.02 m/s) between 2 and 6 weeks after primary TKA, measured using an instrument. Considering a significance level of 0.05, a power (1–β) of 0.80, and a paired *t*-test, as well as adopting a conservative standard deviation of 0.30 m/s for the paired difference, we estimated a minimum required sample size of 14 participants to detect this effect; to accommodate potential loss to follow-up (~25%), the sample was increased to 18 patients.

The inclusion criteria required individuals to present with advanced knee osteoarthritis (grade ≥ 4 according to the Kellgren and Lawrence scale), be aged 50–80 years, and have undergone their first surgery for osteoarthritis via the transquadriceps approach. The exclusion criteria comprised neurodegenerative diseases, secondary osteoarthritis, primary unilateral total arthroplasty via a subvastus approach, prior knee surgeries, a history of knee fracture within the previous 12 months, hip arthroplasty, pre- or postoperative infections, comorbidities that could compromise recovery and rehabilitation, or a history of revision knee arthroplasty.

Two postoperative follow-up intervals were chosen a priori to capture both mid-term stabilization and the long-term sustainability of functional recovery. The first assessment (18 months) corresponded to the period when most patients complete standard rehabilitation, and functional gains typically plateau [15,16]. The second assessment (53 months) was scheduled at approximately 4 years post-surgery to evaluate whether improvements in gait quality and inter-limb symmetry were maintained, declined, or further adapted over time—an interval for which there are minimal instrumented data in the literature [17,18]. These time points also aligned with routine clinical follow-up visits, ensuring the feasibility of recruitment and the consistency of testing conditions.

Assessments were conducted over a maximum of 30 min per day. The participants were evaluated using an IMU (G-Sensor^®^, BTS Bioengineering^®^, Garbagnate Milanese, Italy) during the execution of the Timed Up and Go (TUG) test. This sensor captured and stored 15 variables related to functional mobility parameters. For each patient, the mean of three TUG repetitions was used. Evaluations took place at two time points: in the initial postoperative assessment (mean: 12 months after surgery) and in a second evaluation approximately 2 years after the surgical intervention. At the outset, the participants completed an identification form, and anthropometric data (body weight, height, and body mass index) were recorded.

The TUG test quantifies, in seconds, the time required for an individual to rise from a standard chair (without armrests), walk three meters, perform a turn, return to the starting point, and sit down again [16]. The participants were instructed to perform the test at a self-selected, safe speed to minimize the risk of falling. Two trials were conducted, with the first serving as a familiarization trial.

Data were collected via a portable G-Sensor^®^ (BTS Bioengineering^®^, Garbagnate Milanese, Italy) positioned at the L5 vertebral level. This wireless inertial system, designed for human motion analyses, is controlled by a data recording unit (capable of supporting up to 16 sensors) via ZigBee radio communication. Each sensor measures 62 × 36 × 16 mm; weighs 60 g; and comprises a triaxial accelerometer (±6 g), a triaxial gyroscope (±300°/s), and a triaxial magnetometer (±6 Gauss). The device is calibrated against gravitational acceleration immediately after manufacture. For this study, a single sensor was used, configured at a sampling frequency of 50 Hz. The captured data were transmitted via Bluetooth to a computer and processed with proprietary software (BTS G-STUDIO^®^, version 2.6.12.0), which automatically provided the parameters of interest [19].

Statistical analyses were performed by one of the authors using procedures appropriate for the nature of the data and the study’s longitudinal design. First, the normality of quantitative variables was assessed with the Shapiro–Wilk test, which is recommended for small samples. This procedure determined whether parametric or nonparametric tests would be used for subsequent comparisons.

Descriptive statistics are presented as measures of central tendency (mean and median) and dispersion (standard deviation, minimum, and maximum values). To compare the results between the two evaluation time points—denoted as Collection 1 and Collection 2—the following tests were applied: a paired *t*-test for variables exhibiting a normal distribution to compare means and the Wilcoxon signed-rank test for variables that violated normality assumptions to compare medians or the direction of paired differences.

In addition to statistical significance testing, effect sizes were calculated to quantify the magnitude of the observed differences independently of the *p*-values. Cohen’s d was used for parametric tests, and the nonparametric effect size coefficient r was computed as r = z/√n. Effect magnitudes were classified conventionally as slight, moderate, or significant.

Correlation analyses were also conducted to explore the associations between biomechanical variables, such as time, acceleration, and velocity, during different test phases. Pearson’s correlation coefficient was applied to normally distributed data, and Spearman’s rank correlation was applied to nonparametric data. In all analyses, a 5% significance level (*p* < 0.05) was used.

Data were initially organized in Microsoft Excel spreadsheets, and subsequent statistical analyses were conducted using IBM SPSS Statistics^®^, version 2019. Artificial intelligence (AI) was not used for literature searches or scientific writing.

## 3. Results

An initial cohort of 20 patients was invited to participate in this longitudinal study. Of these, 19 attended an outpatient clinic for a functional evaluation to determine whether they met the inclusion criteria, and their epidemiological characteristics are detailed in Table 1.

A descriptive analysis of the functional and biomechanical variables revealed the sample’s central parameters at two distinct time points: Collection 1 (18-month postoperative follow-up) and Collection 2 (53-month postoperative follow-up). The evaluated variables included execution time, accelerations during the sit-to-stand and stand-to-sit phases, and velocities and durations of the turning phases (mid-turning and end-turning).

Regarding the total task duration, a mean of 14.97 ± 3.48 s was observed in Collection 1 (median 14.72 s), with a slight increase to 15.47 ± 2.93 s in Collection 2 (median 14.76 s). These findings suggest that overall functional mobility performance was stable over time, with no marked changes in the total execution time.

For variables associated with the sit-to-stand transition, the mean time was 1.95 ± 0.46 s in Collection 1, decreasing slightly to 1.76 ± 0.40 s in Collection 2, indicating a possible improvement in task agility. Anteroposterior, mediolateral, and vertical accelerations showed minimal variation between collections, thus maintaining similar profiles—for example, anteroposterior acceleration averaged 3.92 ± 1.81 m/s^2^ in Collection 1, compared to 3.78 ± 2.40 m/s^2^ in Collection 2. For the stand-to-sit movement, the mean time was 2.08 ± 0.42 s in Collection 1, rising slightly to 2.30 ± 1.10 s in Collection 2 with increased dispersion, which may reflect greater interindividual variability in the sitting strategy in the later evaluation.

The mid-turning phase demonstrated clinically relevant improvements in velocity. The peak mid-turning velocity increased from 106.44 ± 30.96°/s in Collection 1 to 132.77 ± 30.82°/s in Collection 2, while the mean velocity rose from 65.22 ± 21.54°/s to 68.39 ± 22.07°/s, indicating significant functional gains in turning ability, as supported by the statistical results previously reported.

**Figure 1:**
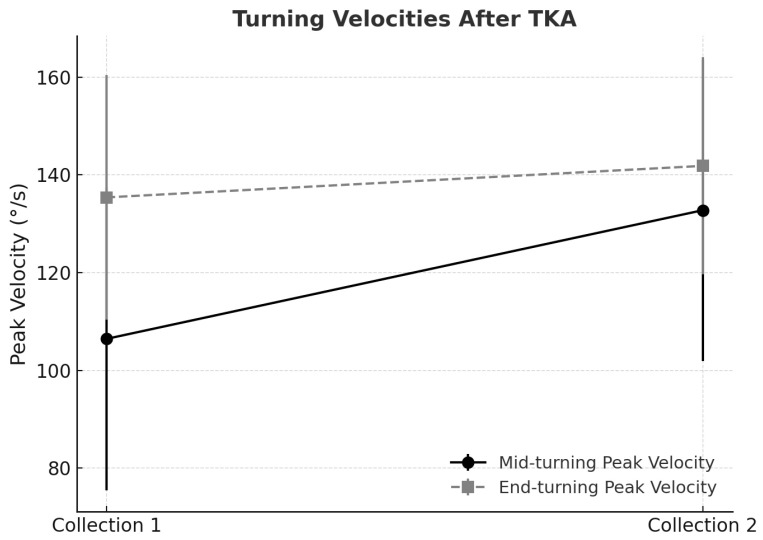
Mid-turning and end-turning velocity changes.

Velocities also increased in the end-turning phase. The peak end-turning velocity rose from 135.40 ± 25.07°/s in Collection 1 to 141.85 ± 22.22°/s in Collection 2, and the mean velocity increased from 67.01 ± 13.81°/s to 76.77 ± 11.93°/s, reflecting enhanced fluidity and motor control at task completion (Figure 1).

Regarding the duration of turning phases (mid-turning and end-turning), the values remained stable between collections, with small average reductions that appeared not to be clinically meaningful but may reflect subtle adjustments in locomotor strategy.

In summary, the descriptive results indicate the maintenance of global task performance over time, coupled with specific gains in turning velocities (mid-turning and end-turning) and modest improvements in transfer tasks (sit-to-stand). This suggests that, even beyond the first postoperative year, patients tend to refine aspects of movement fluidity and dynamic postural control.

### 3.1. Sample Normality

The normality of the data was assessed using the Shapiro–Wilk test, which is considered appropriate for small-to-moderate sample sizes [1]. Quantitative variables from both collections were evaluated, including execution time, accelerations during the sit-to-stand and stand-to-sit phases, and velocities in the mid-turning and end-turning phases. A significance level of 5% (*p* < 0.05) was adopted. According to the Shapiro–Wilk test, the total task duration in Collection 1 (*p* = 0.4511) and vertical acceleration during the sit-to-stand task in Collection 1 (*p* = 0.3222) exhibited normal distributions (*p* > 0.05).

However, the majority of variables did not follow a normal distribution, as was the case for other acceleration and velocity measures in the mid-turning and end-turning phases. A visual inspection of histograms and Q–Q plots corroborated the statistical test results, revealing right-skewed patterns, particularly for the acceleration variables, which were clustered at lower values, with outliers at higher values [2]. Overall, these findings indicate a mixture of normally and non-normally distributed variables in the sample, justifying the use of nonparametric tests for most of the comparative analyses.

### 3.2. Statistical Test to Assess Significance Between Variables

A comparison between Collection 1 (18 months postoperatively) and Collection 2 (53 months postoperatively) was conducted based on the assessment of data normality, which guided the selection of the statistical tests. Table 2 presents the results of the Shapiro–Wilk normality test for each variable in both collections, as well as the results of the paired comparison tests.

It was observed that only the variable related to the peak velocity during mid-turning (intermediate turn) showed a statistically significant difference between the collections (*p* = 0.0039). This finding indicates a significant improvement in the patients’ performance when executing turns, suggesting gains in agility, stability, and motor control—key factors for functionality in daily living activities.

By contrast, the total task duration (*p* = 0.5825), sit-to-stand duration (*p* = 0.1505), sit-to-stand anteroposterior acceleration (*p* = 0.9826), and peak velocity during end-turning (*p* = 0.230) did not exhibit statistically significant differences between evaluations. Nevertheless, the mean values and trend plots indicate a tendency toward clinical improvement—particularly in postural transition tasks and final turns—even though this did not reach statistical significance.

These findings support the hypothesis that, over a multi-year postoperative period, patients achieve greater improvements in turning-phase gait metrics (mid-turning and end-turning velocities) than in transition-phase metrics (sit-to-stand and stand-to-sit durations).

### 3.3. Spearman’s Correlation Analysis

Spearman’s correlation was chosen due to the nonparametric nature of most variables, as indicated by the Shapiro–Wilk tests. This monotonic correlation coefficient (ρ) is less affected by non-normality and outliers, allowing for an assessment of consistent relationships among the temporal, acceleration, and velocity domains without assuming strict linearity. In the relationship between global performance (total duration) and postural transfers, a moderate positive correlation was observed in Collection 2 (53 months) between the total duration and sit-to-stand duration (ρ = 0.54), suggesting that slower individuals also generally took longer to complete the sit-to-stand phase in this later stage of recovery.

Conversely, in Collection 1 (18 months), anteroposterior acceleration during the sit-to-stand maneuver showed a moderate negative correlation with the total duration (ρ = −0.58), indicating that patients who could generate greater initial leg force completed the entire test more quickly. An analysis of the relationship between transfer acceleration and turning velocity revealed a moderate positive correlation in Collection 1 between anteroposterior acceleration in the sit-to-stand maneuver and the peak mid-turning velocity (ρ = 0.35), suggesting that the ability to develop momentum when rising may improve dynamic control and speed during intermediate direction changes.

Furthermore, in Collection 2 (53 months), the peak velocities during mid-turning and end-turning showed a strong correlation (ρ = 0.60), suggesting that patients with improved agility in one turning phase tended to maintain high performance in the next phase. Lastly, the cross-collection correlations between variables were modest, indicating that functional recovery after knee arthroplasty does not progress uniformly across all parameters. While improvements in acceleration and turning velocity are clear, the stability of the total execution time suggests that the qualitative aspects of movement—such as fluidity and postural control during turns—may serve as more sensitive long-term recovery indicators than overall speed.

### 3.4. Effect Size Measures

Cohen’s d was used for paired *t*-tests, and effect size r (Z/√n) was used for Wilcoxon tests of Collections 1 and 2. The thresholds for interpretation were ≤0.20 (minimal effect), 0.20–0.50 (small effect), 0.50–0.80 (moderate effect), and >0.80 (large effect). The results show that the peak velocity during mid-turning had a significant impact (r = 0.88), confirming the statistical significance observed (*p* = 0.0039) and indicating a substantial improvement in agility and motor control during intermediate turns.

The peak velocity during end-turning showed a moderate effect (d = 0.47), indicating a clinically meaningful improvement in this final turning phase, although it did not reach statistical significance. Transfer variables—both the sit-to-stand duration and anteroposterior acceleration—exhibited minor-to-moderate effects (r ≈ 0.39), suggesting small but noticeable improvements in the postural transition capacity. Lastly, the total test duration remained essentially unchanged (d = −0.13), reinforcing the finding that the overall execution time did not vary significantly between collections.

Taken together, despite the sample size limitation, this effect size analysis shows that the most significant improvements in function occurred in tasks involving changes of direction and dynamic control, which are key for independence in daily activities. Although the overall execution time has consistently been found to be stable in the literature—where qualitative improvements in fluidity and safety may occur before or without changes in the overall task time—the moderate-to-significant effects observed in turning phases highlight the importance of including rotation- and balance-specific exercises in medium- and long-term rehabilitation plans.

The evaluation of turning velocities following unilateral total knee arthroplasty revealed distinct patterns when statistical significance was interpreted alongside the Minimal Clinically Important Difference (MCID). For mid-turning peak velocity, the baseline mean was 106.4°/s (SD 31.0), increasing to 132.8°/s (SD 30.8) at follow-up, corresponding to a mean gain of 26.4°/s. Using a distribution-based method, the MCID was estimated at approximately 15.5°/s (½ SD). The observed improvement not only achieved statistical significance (*p* = 0.0039) but also clearly exceeded the MCID threshold, thereby supporting its clinical relevance. This finding indicates that patients experienced a perceptible enhancement in agility and motor control during turning tasks, which is likely to translate into improved functional mobility and safety in daily activities.

By contrast, end-turning peak velocity demonstrated a more modest change. The baseline mean was 135.4°/s (SD 25.1), increasing to 141.9°/s (SD 22.2), for a mean gain of 6.5°/s. The calculated MCID for this parameter was approximately 12.5°/s. Although the improvement corresponded to a moderate effect size, it did not surpass the MCID threshold, suggesting that the change may not be clinically meaningful despite the apparent trend. This highlights that not all statistically detectable improvements are of practical significance, reinforcing the value of MCID in distinguishing clinically relevant outcomes.

Taken together, these results emphasize the importance of integrating both statistical and clinical perspectives in the interpretation of functional outcomes after total knee arthroplasty. The clinically meaningful improvement in mid-turning velocity underscores the potential for rehabilitation programs to specifically target rotational control and agility, while the limited clinical impact of end-turning gains suggests that further strategies may be required to elicit meaningful benefits in this phase of mobility.

### 3.5. Test–Retest Reliability and Measurement Error

Pearson’s correlation coefficients (r) between the measurements from Collections 1 and 2 ranged from weak to moderate, with higher values observed for the end-turning phase metrics (r = 0.41; R^2^ = 0.16 for phase duration; r = 0.47; R^2^ = 0.22 for peak rotational velocity). In contrast, the stand-to-sit parameters showed weak-to-negligible correlations (r between −0.25 and 0.12; R^2^ < 0.07). The intraclass correlation coefficients (ICC_2_, two-way absolute agreement) were moderate for the end-turning (ICC_2_ = 0.45–0.49) metrics but low to negligible for the stand-to-sit metrics (ICC_2_ from −0.08 to 0.26), indicating limited reproducibility for several variables. The standard error of measurement (SEM) ranged from 0.43 to 9.22 units, indicating notable imprecision, especially in the end-turning peak rotational velocity. Table 3 reports the coefficients of determination (R^2^) for all significant correlations. Overall, these results suggest that the end-turning metrics are more consistent and less prone to relative imprecision than the stand-to-sit metrics, highlighting the need to improve sensor acquisition and calibration protocols to enhance repeatability and the clinical relevance of instrumented TUG assessments.

## 4. Discussion

This study investigated the progression of functional mobility in patients who underwent unilateral total knee arthroplasty (TKA) through a transquadriciptal approach, assessed at two late postoperative time points (around 18 and 53 months). The main finding was not a change in the total task execution time—which remained consistently around 15 **s** in both assessments—but rather a qualitative improvement in movement. Specifically, a statistically significant, large effect was observed in the peak velocity during the mid-turning phase (r = 0.88; R^2^ = 0.77), along with a clinically relevant, moderate improvement in the end-turning velocity (r = 0.47; R^2^ = 0.22; ICC_2_ = 0.49; SEM = 9.22). These results indicate that, even though functional recovery typically stabilizes within the first year after surgery, ongoing refinements occur in more complex aspects of motor control, such as agility and dynamic stability [18].

The stability of the overall Timed Up and Go (TUG) test duration—with average values of about 15 s in both assessments—matches that in longitudinal studies showing that, although the biggest functional improvements after TKA occur within the first 12 months, recovery can take up to 2 years, after which performance tends to level off [19]. The average time in our sample is higher than reference values for healthy adults of the same age group (usually under 12 s) [20], indicating that functional deficits remain even years after surgery. A recent systematic review confirmed that TKA patients continue to show poorer performance on functional tests than healthy controls in the long term [13]. These findings support the hypothesis that certain aspects of gait performance—particularly those involving turning and dynamic transitions—may continue to improve beyond the first postoperative year, even when the overall test duration remains unchanged [21].

The most notable finding was the significant increase in turning velocities. The peak mid-turning velocity rose substantially, with a large effect size (r = 0.88; R^2^ = 0.77), indicating a strong improvement in directional change ability. Turning tasks are neuromotorically more challenging than straight-line walking, requiring greater postural control, coordination, and motor planning [22]. The late improvement in this measure suggests ongoing motor learning and proprioceptive adaptation. As described by Maiora et al., the TUG test is a valuable tool for assessing complex functional mobility [21]. Over time, patients may develop more efficient and safer strategies for turning, which is crucial for daily functioning and reducing fall risk, which is a persistent issue in the TKA population [23]. The strong positive correlation (ρ = 0.60; R^2^ = 0.36) between the mid- and end-turning velocities in the second assessment supports the idea that patients adopt a more consistent and effective turning strategy.

Analyses of postural transfers, such as the sit-to-stand (STS) maneuver, showed small-to-moderate improvement trends, though not statistically significant. The moderate negative correlation (ρ = −0.58; R^2^ = 0.34) between sit-to-stand anteroposterior acceleration and the overall TUG duration in the first assessment indicates that generating initial momentum was an important factor early on. The weakening of this relationship in the second assessment may reflect a shift in functional strategy: while explosive strength was key initially, fluidity and dynamic control during turns became the main factors influencing long-term performance quality. Importantly, the STS metrics exhibited low test–retest reliability (ICC_2_ = 0.26; SEM = 0.72), suggesting that the observed trends might be within the measurement error and should be interpreted cautiously [23].

Low intraclass correlation coefficient (ICC) values observed for some of the instrumented Timed Up and Go (TUG) measurements in this study warrant careful consideration. Reliability indices such as ICC reflect the consistency of repeated measurements across time, and low values may indicate that variability is not attributable solely to true patient differences, but also to methodological or technical factors. A recent systematic review reported wide variability in intraclass correlation coefficient (ICC) values across iTUG components, ranging from moderate to excellent depending on the segment analyzed and the protocol used, and concluded that the complexity and lack of standardization of extracted measures reduce reliability and validity—particularly for turning and transition phases, where low ICCs were frequently observed [24]. Convergent-validation and test–retest reliability studies corroborate these findings: overall TUG duration and walking phases typically show high reliability (ICC > 0.9), whereas subcomponents such as turning and sit-to-stand/stand-to-sit transitions often exhibit low to moderate ICCs (sometimes < 0.5), implicating technical factors (e.g., segmentation algorithm accuracy and sensor sensitivity) and variability in test execution [25,26]. These results are consistent across diverse populations—including older adults, patients with neurological disorders, and children—highlighting the variable reliability of iTUG subcomponents and the need for greater protocol standardization and improved analysis algorithms [24,25,26,27].

One plausible explanation is measurement error, which can arise from several sources. The inertial measurement unit (IMU) used in this study, although validated for functional mobility assessments, may introduce variability due to signal noise, drift in gyroscopic data, or limitations in the sampling frequency. Small deviations in data acquisition or post-processing can accumulate and influence derived kinematic parameters such as acceleration or turning velocities, and measurement error—originating from signal noise, gyroscopic drift, limited sampling frequency, and small deviations during data acquisition or post-processing—can accumulate and materially affect derived kinematic metrics such as acceleration and turning velocities [28]. Together, these observations underscore the need for methodological refinements (e.g., rigorous sensor calibration, drift-compensation algorithms, adequate sampling rates, standardized segmentation and filtering procedures, and transparent post-processing pipelines) to reduce measurement bias and improve the reliability of sensor-based kinematic outcomes.

Another contributing factor may be sensor placement. The IMU was positioned at the L5 vertebral level, following standard protocols, but slight deviations in placement, orientation, or fixation tightness between sessions could alter the accuracy of captured signals. Given that patients were evaluated months to years apart, minor inconsistencies in sensor setup could have impacted reproducibility. There is broad consensus in the literature that small variations in the position, orientation, or fixation of an inertial measurement unit (IMU)—even when standardized protocols are followed—can introduce systematic errors and compromise data reproducibility, particularly for assessments spaced months or years apart. Rigorous standardization of sensor placement and thorough documentation of attachment procedures are therefore recommended to minimize these effects and to improve reliability in longitudinal and multicenter studies [29,30,31,32].

In addition, patient-related factors may also contribute. Functional tasks such as sit-to-stand or stand-to-sit involve greater variability in strategy across individuals, which may explain why ICC values were lowest for these parameters. Unlike walking or turning—where motor strategies are relatively consistent—transitional movements are more influenced by compensatory behaviors, fatigue, or fear of instability, leading to greater intra-individual variability. Other studies indicate that postural-transition tasks show greater variability—driven by individual differences and compensatory strategies—which is reflected in lower intraclass correlation coefficients (ICCs) for these parameters compared with gait or turning tasks [33,34].

Finally, the small sample size must be considered, as it amplifies the influence of outliers on reliability statistics. In small cohorts, even a few inconsistent trials can markedly reduce ICC values, underestimating the true reproducibility of the instrumented TUG.

Taken together, the low ICC coefficients likely reflect a combination of technical (measurement error, sensor placement), procedural (longitudinal variability in testing conditions), and patient-related (heterogeneity in movement strategies) factors. Future studies should aim to minimize these sources of variability by standardizing sensor placement protocols, increasing the number of test repetitions, and validating outcomes in larger samples to provide more robust reliability estimates.

These findings have important clinical implications. First, evaluating functional recovery after TKA should go beyond simple time-based measures. Analyzing specific aspects of the task—such as turning phases—with technologies such as IMUs provides a more sensitive view of movement quality, especially as end-turning metrics show moderate reliability (ICC_2_ ≈ 0.49; SEM ≈ 9.22) compared to the low reliability of transition phases. Additionally, our results highlight the need for rehabilitation programs that extend beyond the first year after surgery. These programs should include exercises that improve dynamic postural control, coordination, and agility during directional changes, focusing on enhancing performance in daily activities rather than only the linear gait capacity. No studies identified in the analyzed medical literature significantly refute these findings. On the contrary, available data reinforce the importance of using technologies such as IMUs for a more comprehensive and sensitive assessment of functional recovery after TKA, as well as the need for prolonged and targeted rehabilitation for complex daily functional demands [35].

This study has several limitations that must be acknowledged. First, the small sample size (n = 19) limits its statistical power and generalizability. The lack of a control group—either composed of healthy individuals or patients treated with a different surgical approach—limits the generalizability and interpretability of our findings. As such, it is not possible to determine whether the observed changes are directly attributable to the surgical technique itself or reflect natural functional recovery over time. Moreover, relevant confounding factors such as physical activity levels and adherence to physiotherapy protocols were not consistently recorded in the patients’ medical files and were thus not controlled for in this study. However, given the detailed and objective nature of the functional assessments, this case series offers valuable descriptive data to support the design of future comparative studies.

Future research should focus on validating these findings in larger, multicenter cohorts. Comparative studies assessing TUG kinematics after surgery using different approaches (such as transquadriciptal, subvastus, and medial parapatellar approaches) would be especially valuable. Additionally, exploring the relationships between kinematic parameters and other factors—such as quadriceps and hip muscle strength, patient confidence measures, and fall rates—could help clarify the mechanisms behind this late functional improvement.

## 5. Conclusions

This study shows that, in older adults who underwent unilateral total knee arthroplasty through a transquadriciptal approach, overall performance measured using the Timed Up and Go (TUG) test remained stable between 18 and 53 months post-surgery. However, qualitative movement parameters—specifically turning velocities (mid-turning and end-turning)—demonstrated significant and clinically meaningful improvements [1]. The strong increase in the peak mid-turning velocity, along with the moderate effects in end-turning and postural transfer components, suggests a late and ongoing enhancement in movement fluidity and motor control, which are essential for independence and reducing fall risk [2].

These findings highlight the importance of conducting segmented assessments and using IMUs to monitor subtle functional improvements that traditional time-based metrics might miss. It is advised that physiotherapy protocols extend beyond the first postoperative year and include exercises focused on rotational control and balance. Future multicenter studies with comparative groups are essential to validate and build upon these results.

## Figures and Tables

**Table 1:** Demographic and clinical characteristics of the patient sample (*n* = 19).

**Variable**	**Mean ± SD**	**Minimum**	**Maximum**
Age (years)	69.2 ± 6.1	60	82
Height (m)	1.61 ± 0.11	1.40	1.87
Weight (kg)	100.9 ± 20.1	69.3	138.1
BMI (kg/m^2^)	31.3 ± 4.2	24.8	41.2
Postoperative time—Collection 1 (months)	17.3 ± 2.1	15.2	19.4
Postoperative time—Collection 2 (months)	42.8 ± 3.5	39.3	46.3
**Categorical Variable**	***n* (%)**		
Right limb	14 (73.7%)		
Left limb	5 (26.3%)		

Legend: SD: standard deviation; BMI: body mass index; *n*: absolute frequency.

**Table 2:** Comparison of statistical tests between Collection 1 (18 months postop) and Collection 2 (53 months postop).

Variable	Test	Shapiro–Wilk *p* Collection 1	Shapiro–Wilk *p* Collection 2	Test Statistic	*p* Value
**Total Task Duration**	Paired *t*-test	0.4511	0.1128	−0.5598	0.5825
**Sit-to-Stand Duration**	Wilcoxon signed-rank test	0.0391	0.7071	52.5	0.1505
**Sit-to-Stand Anteroposterior Acceleration**	Wilcoxon signed-rank test	0.0409	0.0001	85.0	0.9826
**Mid-Turning Peak Velocity**	Wilcoxon signed-rank test	0.0022	0.2905	26.0	0.0039
**End-Turning Peak Velocity**	Paired *t*-test	0.6651	–	−1.2425	0.2300

**Table 3:** Coefficients of determination (R^2^) for all significant correlations.

Variable	r	R^2^	ICC_2_	SEM
End-Turning	0.4061	0.1649	0.4505	0.4313
End-Turning 2	0.4657	0.2169	0.4900	9.2197
Stand-to-Sit.1	−0.2546	0.0648	−0.0771	0.9924
Stand-to-Sit.2	−0.2202	0.0485	−0.0347	1.2127
Stand-to-Sit	0.1229	0.0151	0.2580	0.7221

## Data Availability

The raw data supporting the conclusions of this article will be made available by the authors on request.

## Abbreviations

The following abbreviations are used in this manuscript:

G-Sensor	Wearable inertial measurement unit (BTS Bioengineering, Italy)
L5	Fifth lumbar vertebra (sensor placement level)
SPSS	Statistical Package for the Social Sciences (IBM SPSS Statistics)
CAAE	Certificado de Apresentação para Apreciação Ética (Brazilian ethics approval certificate)
SUS	Sistema Único de Saúde (Brazilian Unified Health System)
IA	Inteligência Artificial (artificial intelligence)
